# Impact of Inflammatory Immune Dysfunction in Psoriasis Patients at Risk for COVID-19

**DOI:** 10.3390/vaccines9050478

**Published:** 2021-05-10

**Authors:** Tatiana Mina Yendo, Maria Notomi Sato, Anna Cláudia Calvielli Castelo Branco, Anna Julia Pietrobon, Franciane Mouradian Emidio Teixeira, Yasmim Álefe Leuzzi Ramos, Ricardo Wesley Alberca, Cesar Giudice Valêncio, Vivian Nunes Arruda, Ricardo Romiti, Marcelo Arnone, André Luis da Silva Hirayama, Alberto Jose da Silva Duarte, Valeria Aoki, Raquel Leao Orfali

**Affiliations:** 1 Department of Dermatology, Faculdade de Medicina FMUSP, Universidade de Sao Paulo, 01246-903 Sao Paulo, Brazil; tatiana.yendo@hc.fm.usp.br (T.M.Y.); marisato@usp.br (M.N.S.); cesar.valencio@hc.fm.usp.br (C.G.V.); vivian.arruda@hc.fm.usp.br (V.N.A.); r.romiti@hc.fm.usp.br (R.R.); marcelo.arnone@hc.fm.usp.br (M.A.); andre.hirayama@hc.fm.usp.br (A.L.d.S.H.); alberto.duarte@hc.fm.usp.br (A.J.d.S.D.); valeria.aoki@hc.fm.usp.br (V.A.); 2 Laboratory of Dermatology and Immunodeficiencies (LIM-56), Department of Dermatology, Faculdade de Medicina FMUSP, Universidade de Sao Paulo, 01246-903 Sao Paulo, Brazil; annabranco@usp.br (A.C.C.C.B.); pietrobonaj@usp.br (A.J.P.); franciane.mteixeira@usp.br (F.M.E.T.); yasmim.leuzzi@usp.br (Y.Á.L.R.); ricardowesley@usp.br (R.W.A.)

**Keywords:** psoriasis, COVID-19, SARS-COV-2, IL-27, immune response, cytokines

## Abstract

Psoriasis is an immune-mediated dermatosis usually associated with comorbidities. Treatment varies from topicals to systemic drugs and data on susceptibility to viral infections in psoriatic patients are scarce. The objectives of this study were to analyze psoriatic patients on different therapies who were at risk for COVID-19 for seroprevalence of SARS-COV-2, pro-inflammatory cytokine profile, comorbidities and outcomes in order to unveil the immunological mechanisms involved in the anti-viral response in patients with psoriasis. Seventy-five patients with psoriasis were divided according to treatment: immunobiologics, methotrexate, topicals and acitretin. Twenty healthy controls were included. Plasma samples were collected for: IgG SARS-COV-2 (ELISA); IL-27, IL-29 and IL-18 (ELISA); and IL-1β, IL-17A, IL-6 and TNF (cytometric array). Seropositivity for SARS-COV-2 was detected in 24 out of 75 psoriasis patients and did not relate to COVID-19 symptoms and/or hospitalization, despite associated comorbidities. Psoriasis patients who were asymptomatic for SARS-COV-2 exhibited immune imbalance with high levels of IL-18, IL-17A and IL-6, and low levels of IL-27 compared to healthy controls. Psoriasis groups showed significant increased cytokine levels only in the group with immunobiologics. Despite immune deviations and lower IL-27, which has a potential antiviral impact, psoriatic patients did not exhibit complications related to COVID-19. An understanding of this kind of proinflammatory profile of psoriatic patients and of the lack of severe outcomes for COVID-19 is essential to establish novel therapeutic approaches and preventive measures, including with regard to the concomitance of viral infections.

## 1. Introduction

Psoriasis is a chronic and inflammatory immune-mediated skin disease featuring genetic predisposition and associated comorbidities (psoriatic arthritis, cardiovascular disease, obesity, hypertension, dyslipidemia, diabetes mellitus, non-alcoholic fatty liver disease, inflammatory bowel disease, depression, and lymphoma, among others), with a worldwide prevalence of around 2% and a high impact on quality of life [1,2,3]. Psoriasis lesions are characterized by scaly erythematous plaques, which are presented in different phenotypes (psoriasis vulgaris, palmoplantar psoriasis, erythrodermic psoriasis, inverse psoriasis, guttate psoriasis and palmoplantar psoriasis) and with various degrees of severity. Individual susceptibility and environmental factors have key roles in determining the disease presentation. Medication—such as beta-blockers, lithium and anti-malarial and non-steroidal anti-inflammatory drugs—emotional distress, physical trauma, and infections are examples of external factors that may elicit or aggravate psoriasis in a genetically susceptible individual by breaking immunologic tolerance. Histological features of this condition include parakeratosis (due to hyperproliferative keratinocytes in the basal membrane of the epidermis), regular acanthosis, intraepidermal micro-abscesses containing neutrophils, dilatated capillaries on the dermal papillae and a dermal inflammatory infiltrate composed of lymphocytes, histiocytes, neutrophils and dendritic cells [4].

When the disease is not under control, patients are in a chronic inflammatory state with elevated serum inflammatory markers. The pathogenesis of psoriasis was initially related to a predominant Th1 axis, as patients with psoriasis present high levels of IFN-γ−producing Th1 lymphocytes in cutaneous lesions and in the peripheral blood [5]. Nowadays, the pathogenesis of psoriasis is mainly considered to be mediated by Th17, as a crucial part of psoriasis skin proliferation and inflammation, and endorsed by IL-23 [6,7]. Both innate and adaptive cutaneous immune responses are involved in skin psoriatic inflammation, in which cutaneous damage leads to keratinocytes death and DNA and RNA release in the extracellular environment. Plasmacytoid dendritic cell activation—mediated by human cathelicidin antimicrobial peptide (CAMP), known as LL-37, and Toll-like receptor 9 (TLR9)—complexed to self-nucleotides exerts a key role in the disease, leading to production of type I IFN (IFN-α and IFN-β) as well as Th1 and Th17 cells interleukins (IFN-γ and IL-17, respectively) and proinflammatory cytokines (TNF-α, IL-12, IL-23, IL-6 and IL-1β). Other pro-inflammatory cytokines—such as IL-23, which is produced by dermal leucocytes from a myeloid lineage —induce the proliferation and differentiation of Th17 lymphocytes and perpetuate the inflammatory cascade in psoriasis [1,7,8,9].

The IL-23/Th-17 pathway also induces IL-29 (a type III IFN—IFNλ) secretion, which is responsible for stimulating the release of antiviral proteins by keratinocytes, mediating an antiviral state on psoriatic skin [2,7]. IL-27, a member of the IL-6/IL-12 family (a heterodimer composed of p28 and Epstein–Barr virus-induced 3—EBI3), also induces antiviral proteins in an IFN-independent way and can affect both induction and/or inhibition of inflammation [10,11]. IL-27 inhibits Th17 cells differentiation and exhibits an anti-inflammatory function in Th cells. In psoriasis, IL-27 can both be responsible for disease onset itself, inducing Th1 lymphocytes, and can suppress the inflammation when TNF-α is upregulated, suggesting that manipulation of IL-27 in psoriasis could be beneficent [6,7,10].

IL-27 is also capable of regulating IL-18 binding protein (BP) in skin resident cells [12]. IL-18, a member of the IL-1 family, mediates either Th1 or Th2 cell responses and acts as an important regulator for both innate and acquired immunity. IL-18 also demonstrates an important proinflammatory property in skin-tropic viruses by either producing a viral antagonist (vIL-18BP) or inducing endogenous IL-18BP secretion [12,13]. In psoriasis, circulating levels of IL-18 are enhanced.

The choice of psoriasis treatment is based on the severity and extension of the disease, the topography of the cutaneous lesions, the associated comorbidities, the impact on quality of life, the availability of medications, the adverse effects profile and the clinical and social background of the patient. Topical treatment is an option for mild and localized disease. Topical steroids, topical calcineurin inhibitors, topical vitamin D analogues and keratolytic agents are examples of medications used for psoriasis. Classic systemic treatments comprise phototherapy, immunosuppressants (methotrexate and cyclosporine for instance) and retinoic acid (acitretin) and they are indicated for severe or recalcitrant psoriasis. Novel treatments with biologicals agents, such as anti-TNFα, anti-IL17, anti-IL12 and IL-23, are highly effective, but their prescription is onerous for a public health system [14].

In this study, we aimed to analyze asymptomatic psoriasis patients at risk for COVID-19 treated in our university hospital, which is a tertiary public healthcare facility that became a referral hospital for severe SARS-CoV-2 infected patients after the beginning of the COVID-19 pandemic. Those patients were under different therapies and were evaluated for seroprevalence of SARS-COV-2, pro-inflammatory cytokine profile comorbidities and outcomes. Our goal was to evaluate the inflammatory profile of patients with psoriasis under different treatment regimens who were asymptomatic for COVID-19 infection and highly exposed at a referral health facility for SARS-CoV-2. These findings may help in the development of further strategies to adapt anti-inflammatory/immunosuppressant treatment for psoriasis during the COVID-19 pandemic.

## 2. Materials and Methods

### 2.1. Subjects

Seventy-five patients with psoriasis (Pso) at risk for COVID-19 and 20 healthy controls (HC, *n* = 20) were selected. All patients were from the Outpatient Psoriasis Clinic, Dermatology Department, Hospital das Clínicas, Faculdade de Medicina da Universidade de Sao Paulo (FMUSP), Sao Paulo, SP, BR. The Pso patients were divided into four groups according to treatment option: group 1 (G1)—immunobiologics (*n* = 20); G2—methotrexate (*n* = 20); G3—topicals (*n* = 15); G4—acitretin (*n* = 20); blood samples were collected during the pandemic phase of COVID-19 (from July to August 2020). No Pso patient discontinued treatment and they all agreed to fill out a questionnaire regarding associated comorbidities, COVID-19 symptoms and social isolation. All individuals read the statement of informed consent and agreed to participate. The study was approved by the local Ethics Committee. Methods were performed in accordance with the relevant guidelines and regulations of this institution. Demographic data are shown in Table 1.

### 2.2. Isolation of Plasma Samples

Venous blood samples were collected in EDTA-anticoagulated tubes and centrifuged at 1500 rpm for 5 min for plasma isolation; samples were stored at −80 °C.

### 2.3. ELISA Cytokines Measurement

Plasma samples were quantified for IL-18, IL-27 and IL-29 using DuoSet enzyme-linked immunosorbent assay (ELISA) kits (codes: DY318-05, DY2526 and DY7246, respectively; R&D Systems, Minnesota, MN, USA) according to the manufacturer’s instructions. The minimum detection limits were: IL-18–12 pg/mL; IL-27–156 pg/mL; and IL-29–63 pg/mL.

### 2.4. Detection of IgG Antibodies to COVID-19

Plasma samples from healthy donors and psoriasis patients were tested for the presence of anti-COVID-19 IgG by ELISA using commercial kits from Dia.Pro (Diagnostic Bioprobes Srl, Milan, Italy). The assays were performed according to the manufacturer’s instructions and detected antibodies against a mix of recombinant antigens (nucleocapsid, S1 and S2). The test results were calculated considering the sample reading measures and cut-off values (S/Co), as suggested by the producer. An S/Co <0.9 indicated that the subject had no IgG antibodies to COVID-19 while S/Co values >1.1 were indicative of antibodies against COVID-19.

### 2.5. Determination of Cytokines by Cytometric Bead Array (CBA)

The cytokines IL-6, IL-17A, TNF and IL-1β were quantified in plasma samples with the CBA method using commercial kits (Flex Cytometric Bead Array Enhanced Sensitivity and Flex Cytometric Bead Array; BD Pharmingen, San Diego, CA, USA) according to the manufacturer’s instructions. The analysis was performed using a flow cytometer (LSR Fortessa; BD Biosciences, San Jose, CA, USA) and data analysis was performed using the FCAP Array software (BD Biosciences). The detection limits were: 68.4 fg/mL for IL-6, 26.1 fg/mL for IL-17A, 67.3 fg/mL for TNF and 2.3 pg/mL for IL-1β.

### 2.6. Statistical Analysis

The Mann–Whitney and Kruskal–Wallis tests were utilized to compare two and three sets of data, respectively. Differences between groups were considered statistically significant when *p* < 0.05, determined using GraphPad Prism software version 9.1.0 (San Diego, CA, USA).

## 3. Results

### 3.1. Positivity of IgG Antibodies to COVID-19 in Psoriasis Patients

A total of 24 out of 75 psoriasis patients were IgG COVID-19 positive. The prevalence of seropositivity to COVID-19 was more prominent in G2 (methotrexate group) and G4 (acitretin group). Healthy controls were seronegative for IgG COVID-19 (data summarized in Table 1).

### 3.2. Analysis of Circulating Levels of Proinflammatory Cytokines

**Figure 1:**
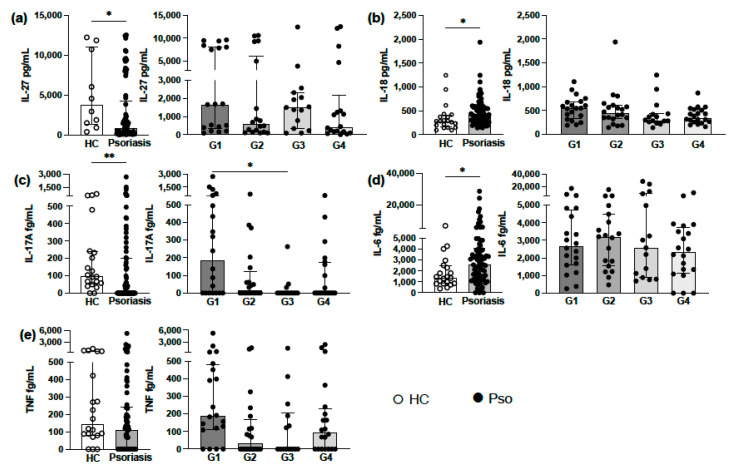
Circulating levels of proinflammatory cytokines in psoriasis. (**a**) Decreased levels of IL-27 in psoriasis (Pso, *n* = 75) compared to healthy controls (HC, *n* = 10). There was no statistical difference between Pso groups. G1 showed more prominent alterations. (**b**–**e**) Increased levels of IL-18, IL-17A, IL-6 and TNF in Pso group (*n* = 75) compared to HC (*n* = 20). When analyzing cytokines levels in Pso groups, we detected increased levels of IL-17A in G1 (**c**). G1—biologics treatment; G2—methotrexate treatment; G3—topical treatment; G4—acitretin treatment. Lines represent medians with the interquartile range of cytokine levels expressed. * *p* < 0.05, ** *p* < 0.01.

In our Pso patients, asymptomatic for SARS-COV-2, we detected an immune imbalance with increased levels of IL-18, IL-17A and IL-6 and decreased levels of IL-27 compared to healthy controls. With regard to the analysis of cytokines levels between psoriasis groups, increased cytokines levels were mainly verified in G1 (Figure 1a–e). We also found that IL-29 was detected in only 9 out of 75 Pso patients and in 1 HC and that IL-1β was detected in 8 out of 75 Pso patients.

### 3.3. Comorbidities in Psoriasis Patients

**Figure 2:**
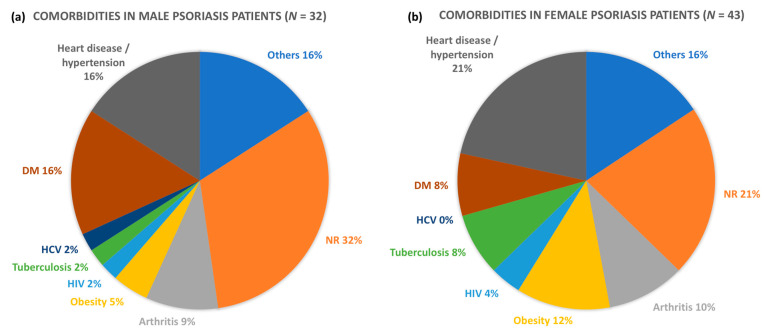
Comorbidities and psoriasis patients. Graphic illustration of main comorbidities reported by male (*n* = 32) (**a**) and female (*n* = 43) (**b**) Pso patients. DM: diabetes mellitus; HCV: hepatitis C virus infection; HIV: human immunodeficiency virus infection; NR: not related.

Figure 2a,b show the main comorbidities reported by the Pso group divided by gender, illustrating the prominence of heart diseases/hypertension, diabetes mellitus, hepatitis C virus (HCV) infection, tuberculosis, human immunodeficiency virus (HIV) infection, obesity and arthritis. Despite associated comorbidities, none of the Pso patients seropositive for COVID-19 (IgG) were hospitalized or showed clinical complications due to SARS-COV-2 infection. Interestingly, diabetes mellitus and HCV infection were more frequent in the questionnaires answered by male psoriasis patients, whilst comorbidities such as heart disease/hypertension, diabetes mellitus, tuberculosis, HIV infection and obesity were more often detected in the questionnaires answered by females.

## 4. Discussion

Psoriasis is a chronic immune-mediated disease with many associated comorbidities. Psoriasis lesions show keratinocyte over-proliferation and atypical infiltration of effector T cells, dendritic cells, natural killer cells, neutrophils and macrophages [15]. This disease is considered by some researchers as a systemic inflammatory condition once it is characterized by a systemic inflammation similar to rheumatoid arthritis [4]. In this study, we evaluated psoriasis patients on different therapies who were at risk for COVID-19 for seropositivity for SARS-COV-2, pro-inflammatory cytokine profile, comorbidities and outcomes.

The COVID-19 seroprevalence in the city of Sao Paulo at the time this study was conducted was 11% [16]. Interestingly, the COVID-19 seroprevalence in our Pso group was higher (32%) compared to the general municipal data. Our outpatient clinic is a public healthcare facility that mainly assists the low-income population. Social and economic disparities are reflected in the access to educational and health systems, thus jeopardizing the least privileged population, who are at greater risk of SARS-COV-2 contamination [17]. Limited access to diagnostic tests, contact tracing and early diagnosis and low isolation rates may explain the higher prevalence of positive serology for COVID-19 in our Pso group.

All Pso patients included in this study were asymptomatic for COVID-19, with a seropositive prevalence of 32% for SARS-COV-2. Incredibly, this prevalence was higher in those patients on methotrexate (G2) and acitretin (G4). None of the psoriatic patients needed hospitalization or suspension of their current medication due to COVID-19 infection, indicating that no harm results from immunobiological treatment in psoriasis patients at risk for COVID-19. Similar findings were reported for a study with 374 patients with psoriasis; hospitalization due to COVID-19 infection was more frequent in patients treated with non-biologic systemic therapy compared to those treated with biologics, which was attributed to the fact that the first group presented lower levels of isolation compared to the latter [18].

During the COVID-19 outbreak, some data led to suggestions of caution in prescribing or sustaining immunosuppressants or immunobiological medications in chronic skin conditions [19,20]. As the pandemic progressed, maintenance of such treatment options proved to be safe, except in cases of hospitalization or complications due to COVID-19 [21,22,23,24,25], which reinforces our findings.

Psoriasis patients exhibit enhanced circulating levels of IL-6, IL-18, IL-17A, IFN-γ, TNF, IL-1β, IL-27 and IL-29 [12,26,27,28]. The inflammatory cytokine profile of Pso patients at risk for COVID-19 included in our study showed increased levels of IL-18, IL-17A and IL-6 and decreased levels of IL-27 when compared to HCs. IL-29 was detected in 9 out of 75 and IL-1β in 8 out of 75 of Pso patients. When analyzing cytokine levels across psoriasis groups, increased cytokines levels were mainly verified in G1 (immunobiological treatment), with a tendency toward elevation in G2 and G4 (systemic treatments for psoriasis). This finding may have been due to the limited number of patients included in our study. Patients on systemic treatments have more severe psoriasis, with increased initial PASI scores and recalcitrance to exclusive topical treatments. It is known that Th17 inflammatory axis activation has a positive correlation with the severity of psoriasis [9]. This fact explains the higher detection of IL-17A in G1, G2 and G4, which was not seen in the topical treatment group (G3) with mild psoriasis.

Our findings indicate that, when the IL-23/Th17 pathway is targeted with systemic treatment, IL-1β, IL-27 and IL-29 decrease in accordance with the PASI score. IL-27 is able to inhibit Th17 differentiation [6,10] and we suggest that the increased levels of IL-17A observed in our Pso patients could have been due, in part, to the decreased levels of IL-27. Even though IL-27 and IL-29 are linked to the instigation of antiviral protein production, our Pso patients did not develop any severe symptoms or complications due to COVID-19, suggesting that other proinflammatory cytokines may contribute to the cytokine milieu balance in psoriasis inflammation and thus that modulation of IL-27 does not lead to an increased risk of virus infection in these patients. Blockage of IL-27 in septic patients leads to reduced mortality and bacterial burden, indicating that modulation of IL-27 may be helpful in infections, but studies regarding psoriasis and viral infections are still scarce [7,10,29].

Pso patients have many associated comorbidities, including obesity, which is generally associated with cardiovascular diseases, dyslipidemia and diabetes mellitus. Interestingly, adipose tissue also produces large amounts of proinflammatory cytokines, including TNF-α, IL-6 and IL-17, contributing to worsening of psoriasis [30]. Our Pso group demonstrated high frequencies of heart disease, diabetes mellitus and obesity, among other comorbidities, despite the absence of COVID-19 symptoms. These comorbidities are also associated with an enhanced risk of COVID-19 complications and/or mortality [8,31]. An international case series demonstrated that the presence of risk factors for SARS-COV-2 infection in psoriatic patients increased the hospitalization rate in this population [18]. The proinflammatory environment caused by psoriasis and metabolic syndrome may contribute to aggravating the cases of COVID-19 infection, leading to an exaggerated systemic inflammatory response that damages different organs (cytokine storm). A larger study including psoriasis patients with symptomatic SARS-COV-2 infection is necessary to determine whether the Th17 polarized immune response along with a chronic pro-inflammatory state are associated with poorer outcomes in COVID-19 infection.

The limitations of our study included the limited sample size and selection bias, as all patients were from the same healthcare service and had similar economic and social backgrounds. We did not evaluate patients with psoriasis who were not undertaking any treatment or who were in phototherapy in this study due to the specificities of our university hospital, which only admits referred patients with severe diseases. This analysis represents the first step in the comprehension of innate and adaptative immune responses against viral infections in a population with an imbalanced immune system. Psoriasis, like many other inflammatory and autoimmune diseases—such as multiple sclerosis, rheumatoid arthritis and inflammatory bowel disease—presents a deviation of T lymphocytes to a Th17 pattern [9]. Although the IL-23/Th-17 pathway leads to the production of anti-viral cytokines in the skin of psoriatic patients (type III IFN) [2,7], it is still unknown whether these molecules play an important role in the prevention of systemic infections, such as COVID-19.

Our data showed a pro-inflammatory dysfunction in psoriasis patients, with decreased anti-viral cytokine levels and increased pro-inflammatory cytokines levels, albeit varying according to treatment options. Those Pso patients at risk for COVID-19 with positive IgG serology for COVID-19 and associated comorbidities did not experience complications associated with SARS-COV-2 or require hospitalization, despite their immunologic alterations. A full understanding of the antiviral host defense in psoriasis is necessary in order to improve new preventive and therapeutic treatment options. Our data provide fundamental insights into innate and adaptive responses in psoriasis with regard to cytokine milieu modulation of inflammation and response to viral infections.

## Figures and Tables

**Table 1:** Characterization of psoriasis patients (clinical-epidemiological features and treatment options).

Groups	Gender/n	Age (Years)/Mean ± SD	PASI/Mean ± SD	Seropositive COVID-19 (IgG)/n (Total)	Treatment Option/n
G1	M/8 F/9	20–80/53.73 ± 13.66	0–24/3.744 ± 3.662	5/20	mmunobiologics - anti-IL-17A: 5/20 - anti-TNF-α: 4/20 - anti-IL-12p40/IL-23: 11/20
G2	M/11 F/9			8/20	Methotrexate: 20/20
G3	M/4 F/11			3/15	Topical treatment: 15/15
G4	M/9 F/11			8/20	Acitretin: 20/20
HC	M/5 F/15	24–49/32.75 ± 6.10	NA	None	NA

PASI: Psoriasis Area and Severity Index; G: group; M: male; F: female; HC: healthy controls; NA: not applicable.

## Data Availability

Data are included in the article and are also available on request from the corresponding author.

## References

[1] Rendon A., Schäkel K. (2019). Psoriasis pathogenesis and treatment. Int. J. Mol. Sci..

[2] Wolk K., Witte K., Witte E., Raftery M., Kokolakis G., Philipp S., Schonrich G., Warszawska K., Kirsch S., Prosch S. (2013). IL-29 is produced by T(H)17 cells and mediates the cutaneous antiviral competence in psoriasis. Sci. Transl. Med..

[3] Hawkes J.E., Yan B.Y., Chan T.C., Krueger J.G. (2018). Discovery of the IL-23/IL-17 signaling pathway and the treatment of psoriasis. J. Immunol..

[4] Nestle F.O., Kaplan D.H., Barker J. (2009). Psoriasis. N. Engl. J. Med..

[5] Lew W., Bowcock A.M., Krueger J.G. (2004). Psoriasis vulgaris: Cutaneous lymphoid tissue supports T-cell activation and ‘Type 1’ inflammatory gene expression. Trends Immunol..

[6] Shibata S., Tada Y., Kanda N., Nashiro K., Kamata M., Karakawa M., Miyagaki T., Kai H., Saeki H., Shirakata Y. (2010). Possible roles of IL-27 in the pathogenesis of psoriasis. J. Investig. Dermatol..

[7] Handfield C., Kwock J., MacLeod A.S. (2018). Innate antiviral immunity in the skin. Trends Immunol..

[8] Radzikowska U., Ding M., Tan G., Zhakparov D., Peng Y., Wawrzyniak P., Wang M., Li S., Morita H., Altunbulakli C. (2020). Distribution of ACE2, CD147, CD26, and other SARS-CoV-2 associated molecules in tissues and immune cells in health and in asthma, COPD, obesity, hypertension, and COVID-19 risk factors. Allergy.

[9] Li B., Huang L., Lv P., Li X., Liu G., Chen Y., Wang Z., Qian X., Shen Y., Li Y. (2020). The role of Th17 cells in psoriasis. Immunol. Res..

[10] Carl J.W., Bai X.-F. (2008). IL27: Its roles in the induction and inhibition of inflammation. Int. J. Clin. Exp. Pathol..

[11] Kwock J.T., Handfield C., Suwanpradid J., Hoang P., McFadden M.J., Labagnara K.F., Floyd L., Shannon J., Uppala R., Sarkar M.K. (2020). IL-27 signaling activates skin cells to induce innate antiviral proteins and protects against Zika virus infection. Sci. Adv..

[12] Wittmann M., Doble R., Bachmann M., Pfeilschifter J., Werfel T., Mühl H. (2012). IL-27 regulates IL-18 binding protein in skin resident cells. PLoS ONE.

[13] Orfali R.L., Sato M.N., Takaoka R., Duarte A.J.S., Rivitti E.A., Aoki V. (2008). Atopic dermatitis in adults: Evaluation of peripheral blood mononuclear cells proliferation response to staphylococcus aureus enterotoxins A and B and analysis of interleukin-18 secretion. Dermatitis.

[14] Armstrong A.W., Read C. (2020). Pathophysiology, clinical presentation, and treatment of psoriasis: A review. JAMA.

[15] Deng Y., Chang C., Lu Q. (2016). The inflammatory response in psoriasis: A comprehensive review. Clin. Rev. Allergy Immunol..

[16] Inquérito Sorológico para SARS-COV-2 Evolução Da Prevalência Da Infecção No Município De Sao Paulo. https://www.prefeitura.sp.gov.br/cidade/secretarias/upload/saude/27_8_2020_PPT_COLETIVA_PREFEITO_%20fase%2004.pdf.

[17] Takian A., Kiani M.M., Khanjankhani K. (2020). COVID-19 and the need to prioritize health equity and social determinants of health. Int. J. Public Health.

[18] Mahil S.K., Dand N., Mason K.J., Yiu Z.Z., Tsakok T., Meynell F., Coker B., McAteer H., Moorhead L., MacKenzie T. (2021). Factors associated with adverse COVID-19 outcomes in patients with psoriasis—Insights from a global registry–based study. J. Allergy Clin. Immunol..

[19] Wang C., Rademaker M., Baker C., Foley P. (2020). COVID-19 and the use of immunomodulatory and biologic agents for severe cutaneous disease: An Australian/New Zealand consensus statement. Australas. J. Dermatol..

[20] Elmas Ö.F., Demirbaş A., Kutlu Ö., Bağcıer F., Metin M.S., Özyurt K., Akdeniz N., Atasoy M., Türsen Ü., Lotti T. (2020). Psoriasis and COVID-19: A narrative review with treatment considerations. Dermatol. Ther..

[21] Rodriguez-Villa Lario A., Vega-Diez D., Gonzalez-Canete M., Polo-Rodriguez I., Piteiro-Bermejo A.B., Herrero-Fernandez M., Arevalo-Serrano J., Trasobares-Marugan L., Medina-Montalvo S. (2020). Patient s perspective: Psychological burden of the COVID-19 pandemic in 146 psoriatic patients treated with biological drugs and small molecules in real clinical practice. J. Dermatolog. Treat..

[22] Lima X., Cueva M., Lopes E., Alora M. (2020). Severe COVID-19 outcomes in patients with psoriasis. J. Eur. Acad. Dermatol. Venereol..

[23] Ricardo J.W., Lipner S.R. (2020). Considerations for safety in the use of systemic medications for psoriasis and atopic dermatitis during the COVID-19 pandemic. Dermatol. Ther..

[24] Gisondi P., Facheris P., Dapavo P., Piaserico S., Conti A., Naldi L., Cazzaniga S., Malagoli P., Costanzo A. (2020). The impact of the COVID-19 pandemic on patients with chronic plaque psoriasis being treated with biological therapy: The Northern Italy experience. Br. J. Dermatol..

[25] Georgakopoulos J.R., Mufti A., Vender R., Yeung J. (2020). Treatment discontinuation and rate of disease transmission in psoriasis patients receiving biologic therapy during the COVID-19 pandemic: A Canadian multicenter retrospective study. J. Am. Acad. Dermatol..

[26] Cardoso P.R.G., Lima E.V.D.A., Lima M.M.D.A., Rêgo M.J.B.D.M., Marques C.D.L., Pitta I.D.R., Duarte A.L.B.P., Pitta M.G.D.R. (2016). Clinical and cytokine profile evaluation in Northeast Brazilian psoriasis plaque-type patients. Eur. Cytokine Netw..

[27] Yang B., Suwanpradid J., Sanchez-Lagunes R., Choi H.W., Hoang P., Wang D., Abraham S.N., MacLeod A.S. (2017). IL-27 facilitates skin wound healing through induction of epidermal proliferation and host defense. J. Investig. Dermatol..

[28] Forouzandeh M., Besen J., Keane R.W., Vaccari J.P.D.R. (2020). The inflammasome signaling proteins ASC and IL-18 as biomarkers of psoriasis. Front. Pharmacol..

[29] Morrow K.N., Coopersmith C.M., Ford M.L. (2019). IL-17, IL-27, and IL-33: A novel axis linked to immunological dysfunction during sepsis. Front. Immunol..

[30] Owczarczyk-Saczonek A., Placek W. (2017). Compounds of psoriasis with obesity and overweight. Postępy Hig. I Med. Dosw..

[31] Watanabe M., Risi R., Tuccinardi D., Baquero C.J., Manfrini S., Gnessi L. (2020). Obesity and SARS-CoV-2: A population to safeguard. Diabetes Metab. Res. Rev..

